# Effect of Rehabilitation Program for Muscle Strength, Balance, and Gait Retraining with Visual Feedback in Older Women with and Without Knee Osteoarthritis: Clinical Trial

**DOI:** 10.3390/jpm15120631

**Published:** 2025-12-18

**Authors:** Tatiane Silva de Souza, Daniel Borges Pereira, Rodrigo Jugue Hagihara, Carolina Tayama Fuzinato, Ana Paula Ribeiro

**Affiliations:** 1 Biomechanics and Musculoskeletal Rehabilitation Laboratory, Health Science Post-Graduate Department, School of Medicine, University Santo Amaro, São Paulo 04829-300, Brazil; tassouza@prof.unisa.br (T.S.d.S.); daniel.borges@estudante.unisa.br (D.B.P.); rodrigojugue@estudante.unisa.br (R.J.H.); 2 Medical Residency, Federal University of Rio Grande, Rio Grande 96203-900, Brazil; ctayama@estudante.unisa.br; 3 Sports Medicine and Physical Therapy Department, School of Medicine, University of Sao Paulo, São Paulo 05508-220, Brazil

**Keywords:** osteoarthritis, pain, exercise, knee, gait, balance, elderly, woman, rehabilitation

## Abstract

**Background:** Therapeutic exercises have gained great prominence due to the benefits shown in the treatment of knee osteoarthritis (OA). However, to date, there is no evidence on the effects of an exercise program combined with balance and gait training with visual feedback. **Objective:** To evaluate the therapeutic effect of an intervention program combining lower-limb muscle strengthening, balance training, and gait exercises with visual feedback on the chronic pain, functional, and biomechanical aspects of older women with and without OA knee. **Methods:** Clinical trials study with stratified allocation based on disease status (two-arm, triple-blind—assessor, interventionist, and data manager, parallel-group). In total, 40 older women were recruited: 20 in the OA knee group (OAG, n = 20) and 20 in the control group (CG, n = 20). The intervention included a muscular resistance training program in the lower limbs, and reactive and proactive balance and gait training associated with visual feedback. Both groups received the same intervention. The primary outcomes were pain measured by the Visual Analogue Scale and the questionnaires Western Ontario and McMaster Universities Osteoarthritis Index and Lequesne Algofunctional Index. The secondary outcomes were the six-minute walk test, the Falls Risk Awareness Questionnaire, the Timed Up and Go Test, plantar load distribution during gait, and patients’ acceptability. **Results:** The intervention was effective in improving pain and increasing functionality in older women with OA knee, as measured pre- and post-intervention, compared to the control, with a moderate to high effect size. Body balance increased in older women with OA, as indicated by perceptions of fall risk and walk-test pre- and post-intervention. During gait, a reduction in plantar load (midfoot and rearfoot areas) was observed pre- and post-intervention in OAG compared to the CG. Both groups showed excellent acceptability, suitability, and feasibility of the intervention program. **Conclusions:** The intervention protocol was effective over 2 consecutive months in reducing pain and increasing knee functionality, balance, walking distance, and perception of falls in older women with OA of the knee compared with women without the condition. During gait, when visual feedback was combined with the intervention protocol, it promoted a better distribution of plantar load over the midfoot and the medial and lateral rearfoot regions in older women with knee OA. Clinical Trial: ReBEC (RBR-5w67pz4). Ethics Committee approval (number: 4.091.004).

## 1. Introduction

The older population aged over 60 years has been increasing worldwide, with an estimated increase to approximately 1.2 billion older people by the year 2025, and may progress to 2 billion by the year 2050 [1]. It is estimated that by 2025, falls will account for roughly 150,000 hospital admissions in Brazil, with associated healthcare expenses reaching approximately R$260 million [2].

Studies have shown that 85% of older adults have at least one chronic disease resulting from mobility difficulties and problems arising from joint, bone, and muscle systems [3,4,5,6]. According to the World Health Organization (WHO), osteoarthritis (OA) is a chronic condition and the fourth leading cause of disability among older women, with the greatest share of its societal burden arising from hip and knee involvement [7]. This scenario represents a significant public health challenge, as aging is associated with several musculoskeletal changes, including a gradual decline in muscle mass and strength leading to sarcopenia [3,6,7,8,9], reduced muscle flexibility [3,8,9], impaired postural balance with an increased risk of falls [2], and functional limitations affecting gait and daily living activities [10,11]. These are associated with high economic costs, reaching a percentage of 2.3% from 2019 to 2021, referring to medical and hospital expenses of patients, family members, and health policy management bodies [2,12].

OA is defined as a chronic degenerative disease, marked by the progressive breakdown of articular cartilage, resulting in substantial physical and functional impairment in older adults [7]. Epidemiological data indicate that OA is present in over 80% of the older population aged between 65 and 70 years [7,13], with the highest worldwide prevalence in women (18–20%) compared to men (9.6%) [14]. Among the joints affected by OA, the knee is one of the most frequently involved, commonly presenting with pain and progressive loss of articular cartilage [15,16]. Its etiology is multifactorial, encompassing biochemical, metabolic, and morphological alterations [17,18]. These changes give rise to a characteristic clinical presentation, consisting of pain, swelling, joint stiffness on movement, the formation of osteophytes [7,18], quadriceps weakness, and sensorimotor losses of the plantar base that compromise the balance and gait of older adults [8,9,10,19,20].

To restore the musculoskeletal alterations resulting from OA in older adults, especially in the knee joint, conservative clinical treatment is directed and guided by the American and Brazilian Rheumatology Societies, with the purpose of reducing and relieving pain symptoms, reducing knee joint overload, and increasing or improving the patient’s functional activities, as well as preventing or delaying loss of quadriceps muscle strength and proprioceptive deficits in the plantar support base [21,22,23,24]. According to the evidence in the literature aimed at the conservative treatment of OA knee, therapeutic exercises have gained great prominence, given their benefits, namely: muscle strength exercise training for the quadriceps combined with joint mobility exercises of the lower limbs [21,22,23,25], gait training associated with static balance exercises with visual feedback stimulus [26,27], gait training alone [28,29,30], as well as gait training associated with visual feedback stimulation [31].

Scientific evidence suggests the importance of balance training with proactive, or anticipatory, training, which focuses on preparing the body for potential instabilities before a destabilizing movement occurs. It is primarily based on feedforward (or anticipatory) control. The central nervous system (CNS) uses past experiences and sensory information (visual, somatosensory, vestibular) to predict imminent destabilization. Anticipatory postural adjustments (APAs) are generated to maintain stability during a voluntary movement. Repetitive training strengthens the neural pathways that allow for the rapid activation of trunk and leg muscles before movement. This results in greater efficiency in muscle coordination and the ability to initiate more stable movement patterns. The CNS learns to choose more effective muscle synergies based on practice [32]. Reactive training aims at the ability to recover a stable position after an unexpected disturbance. It is based on feedback (or reaction) control. When an external disturbance occurs (e.g., a push, a slip), the body receives sensory feedback about the loss of balance. The CNS processes this information rapidly and sends commands to the muscles in order to generate recovery responses, such as steps or quick adjustments of the limbs, to avoid falling. This training improves reflexes and the speed of muscle responses. Repeated exposure to unexpected disturbances trains the CNS to select faster and more effective recovery strategies, improving sensory integration and reactive muscle activation [32].

Systematic review studies have shown that lower limb muscle strength training associated with aerobic exercises over a period of 8–12 weeks (3–5 weekly sessions lasting 1 h each session) is effective in reducing knee pain, improving functional capacity, enhancing balance, and lowering the risk of falls [26,27,28,29,30,31,32,33]. According to Vicent et al. [25], older people with OA knees who performed lower limb muscle strength exercises (concentric and eccentric) during a period of 4 months, twice a week, obtained positive results in reducing pain and improvement in muscle strength. Another clinical trial study also in older people with OA knee, performed muscle resistance training associated with balance training for a period of 2 months, 2 times a week. The authors reported improvement in muscle function and strength of the lower limbs, as well as reduced pain and risk of falls [34].

In addition to studies aimed at improving pain and functional disability, some studies have shown beneficial effects of treadmill-adapted gait training in older adults with and without OA knee to reduce the knee abduction moment (KAM) [28,29,30] and the redistribution of plantar pressure [28]. Studies involving clinical trials with gait training associated with visual feedback in older adults with OA knee showed efficiency in reducing KAM and muscle strength of the gastrocnemius, hamstrings, and quadriceps muscles [25,35,36,37]. Other clinical trials using gait training with different feedback modalities (real-time mirror feedback or video-based feedback) have also reported improvements in gait performance, with real-time mirror feedback showing particularly notable effects [31,32]. In addition to gait, study carried out by Oungphalachai et al. [27] about the effects of training with visual feedback for balance and muscle strength of the lower limbs, for one month, three times a week, revealed an increase in static balance and dynamics, but not muscle strength, in which the authors emphasize the short period of time for functional improvement.

Considering the proven benefits of training with muscle strength, balance, and gait exercises with visual feedback in older people with and without OA knee, a combination of exercises with the stimulus of visual feedback in a single protocol to address the limitations of the characteristic clinical framework of the disease has not yet been observed in the literature. This combination may enhance reductions in pain, improve knee and ankle–foot muscle strength, and optimize plantar load distribution, while also contributing to better functional performance and balance. Such effects could help slow disease progression—an especially relevant consideration during the SARS-CoV-2 pandemic, when social isolation measures may exacerbate OA-related decline.

Thus, the objective of this study was to investigate the effects of an intervention program combining lower-limb muscle strengthening, balance training, and gait exercises with visual feedback on the chronic pain, functional, and biomechanical aspects of older women with and without OA knee. We hypothesized that completing the two-month program, performed twice weekly, would lead to improvements in pain, muscle strength, and knee function, as well as enhanced balance and gait performance—with reduced plantar load—in older women with knee OA compared with age-matched controls without the disease.

## 2. Materials and Methods

### 2.1. Study Design and Participants

This is a controlled clinical trial with stratified allocation based on disease status (two-arm, triple-blind—assessor, interventionist, and data manager, parallel-group).

**Figure 1:**
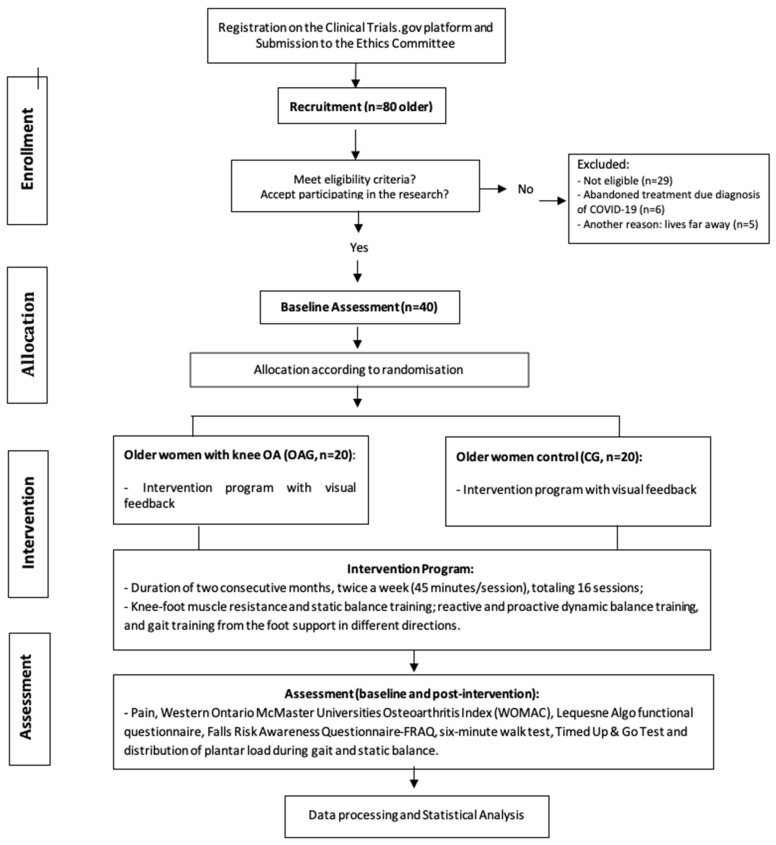
CONSORT: Flow diagram of the clinical trials.

The protocol follows the recommendations of the Standard Protocol Items: Recommendations for Interventional Trials (SPIRIT) and the Consolidated Standards of Reporting Trials (CONSORT) guidelines (Figure 1), and was registered on the clinical trials platform (RBR-5w67pz4). Ethical approval was obtained from the Research Ethics Committee of Universidade local under protocol number 4.091.004.

Forty eligible older women provided written informed consent and were allocated into two groups: twenty with clinically diagnosed medial-compartment knee osteoarthritis (OAG; n = 20) and twenty without the condition, serving as the control group (CG; n = 20). Both groups completed the same intervention protocol, which included lower-limb resistance training (knees and feet), static and dynamic balance exercises, and gait training with visual feedback. All sessions were supervised by a physical therapist and conducted with individualized attention, following all COVID-19 safety measures, including the use of masks, face shields, gloves, and hand sanitizer. Participants were also required to wear masks and use hand sanitizer throughout the trial. Both groups continued their usual care as recommended by their healthcare providers, including pharmacological management and self-care guidance [38].

The older women in both groups were evaluated at two moments: at the beginning of the study (T0) and at the end of the two consecutive months of the intervention (T8). The sessions were held twice a week, totaling 16 treatment sessions of the intervention protocol. During the intervention protocol, all older women received a notebook and instructions to record the use of painkillers on the knee when necessary to manage pain at home. At the end of the study, all subjects received an educational kit on the benefits of knee exercises and guidance on carrying out daily activities at home [38].

Eligibility criteria included: age between 60 and 80 years; clinical and radiographic diagnosis of knee OA according to the American College of Rheumatology criteria; medial-compartment knee OA classified as grades II or III on the Kellgren–Lawrence scale; knee pain between 30 and 80 mm on the visual analogue scale, to reduce baseline variability and avoid pain overestimation; and a body mass index (BMI) < 35 kg/m^2^. Exclusion criteria were: isolated lateral-compartment knee OA (grades II–III), due to distinct biomechanical characteristics; asymptomatic knee OA in one or both knees; use of assistive devices or any lower-limb orthoses (including insoles); physical therapy, acupuncture, or other physical treatments within the previous three months or during the intervention; intra-articular corticosteroid or hyaluronic acid injections within the last 3 and 6 months, respectively; history of hip, knee, or ankle surgery within the past two years; neurological disorders; diabetic neuropathy; rheumatoid arthritis; severe varus or valgus deformities requiring assistive devices; inability to walk independently; and any changes in pharmacological treatment during the study period [38].

Eligibility screening, informed consent procedures, data collection, and statistical analyses were conducted by researchers blinded to group allocation. All participants received oral and written information regarding the study’s risks and benefits and provided written informed consent prior to participation.

### 2.2. Sample Size

The sample size was calculated to detect differences between the intervention program—comprising muscular endurance, balance, and gait exercises with visual feedback—and the standard care recommended by the Osteoarthritis Research Society International (OARSI) clinical trial guidelines. The calculation was based on the mean pre-to-post change in the WOMAC pain subscale. Previous studies report a minimal clinically important difference (MCID) of 1.5–2.0 points on the WOMAC pain scale for individuals with knee OA, with an estimated standard deviation of 2.0–2.5 points [38]. Using these parameters, with an alpha of 0.05 and 80% statistical power, we determined that a total of 40 participants (20 per group) would be sufficient to detect a clinically meaningful difference. A 15% dropout rate was also considered in this calculation. Although participant attrition occurred during the COVID-19 pandemic, both intention-to-treat and per-protocol analyses were employed to mitigate the impact of missing data. A post hoc power analysis performed using the final sample size and the observed effect size for the WOMAC pain outcome indicated that the study still achieved 80% power at α = 0.05, confirming that the final sample retained adequate statistical power to detect clinically meaningful differences.

### 2.3. Setting and Recruitment

Patients were recruited (study start: January 2021; end date: december 2022) by convenience sampling (not probability) through announcements and waiting lists from local and regional orthopedic and rheumatology outpatient clinics in the southern region of São Paulo/SP, as well as from the rheumatology outpatient service and the Biomechanics and Musculoskeletal Rehabilitation Laboratory of the local School of Medicine. Potential participants were identified by the project manager and a research assistant. A trained researcher conducted initial telephone screenings to verify eligibility and coordinated all subsequent contacts related to treatment scheduling, assessments, monitoring, and data collection.

### 2.4. Allocation and Blinding

Elderly women were first classified into one of two diagnostic groups—OA or Control—based on clinical and radiological evaluations performed by the attending physician. This preliminary diagnostic stratification was essential, as the research question required a comparison between individuals with and without knee osteoarthritis. Because diagnostic status cannot be altered or randomized, conventional randomization of all participants into a single undifferentiated pool was not feasible.

Thus, participants were allocated to the intervention or control arms by diagnostic stratification, using a computer-generated list with permuted blocks (sizes 2 × 2), created by an independent researcher who had no involvement in recruitment or assessment. Allocation concealment was ensured by using opaque, sealed, and sequentially numbered envelopes, stored securely and opened only at the time of assignment.

Blinding procedures were rigorously implemented. All clinical, functional, and biomechanical assessments were conducted by an evaluator who was blinded to intervention allocation. Likewise, the physiotherapist responsible for administering the intervention was blinded to the participants’ diagnostic group (OA or Control) and received participants solely according to the allocation indicated in the concealed envelopes delivered to the participants. Participants were also instructed not to disclose their diagnosis or allocation status during assessments. All evaluations and intervention sessions were performed individually, and personal data were coded to maintain confidentiality throughout the study.

The context of the COVID-19 pandemic further facilitated the maintenance of blinding during exercise sessions. Each participant was evaluated and treated individually in a controlled clinical environment, with only the participant and the physiotherapist present. Mandatory safety measures—including the use of face masks, hand sanitization, physical distancing, and restricted contact among participants—minimized interactions and reduced the likelihood of unintentional disclosure of group status.

Through this combination of diagnostic stratification, concealed allocation within strata, and strict operational blinding procedures, the study ensured robust blinding of the assessor, the interventionist, and the data analyst, supporting its classification as a controlled clinical study with stratified allocation and triple blinding (evaluator, interventionist, and data manager).

### 2.5. Intervention Program

The intervention was conducted in the Biomechanical Assessment and Musculoskeletal Rehabilitation Laboratory. A physical therapist (researcher 3) supervised all sessions, which followed established progression criteria over a two-month period, with two sessions per week lasting 45 min each, in accordance with American College of Rheumatology guidelines for OA management [27]. Participants were evaluated at baseline and immediately after completing the two-month intervention. Following the exercise program, they were monitored for an additional two months.

The intervention program consisted of three progressive phases: (1) knee–foot muscular resistance training and static balance exercises; (2) reactive and proactive dynamic balance training targeting sensory and motor components; and (3) gait training with visual feedback of foot loading in multiple directions. The intervention was delivered over two consecutive months, twice weekly, totaling 16 sessions. Patients were evaluated at the beginning (T0) and at the end of the intervention, after two consecutive months (T8) [38]. The term “reactive and proactive balance” refers to an individual’s need to combine the ability to anticipate and prevent problems arising from events or tasks (proactivity) with the anticipatory adjustment to respond quickly to unexpected events (reactivity) [38].

All intervention phases were performed barefoot, without conventional footwear, to avoid influencing clinical, functional, or biomechanical outcomes. It is important to note that all exercises included in the intervention program were grounded in evidence-based recommendations from the literature [25,26,27,37,38,39,40], as well as the use of visual feedback (screen projection on peak pressure—real-time kinematic parameter—and foot rolling for self-correction during gait was standardized for all participants) [27,31,32]. At each stage of the intervention protocol, hygiene parameters were carried out with alcohol gel in the laboratory and treatment environment, as well as on the hands of the older women and the team of physical therapists, maintaining the use of a mask throughout the intervention period.

**Figure 2:**
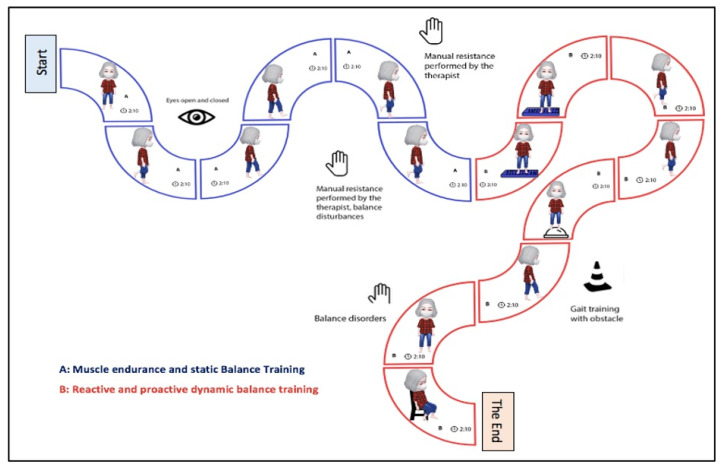
Intervention protocol steps and duration time in older women with and without knee osteoarthritis (OA). (**A**) Muscular resistance and static balance training. (**B**) Reactive and pro-active dynamic balance training (sensory and motor).

**Figure 3:**
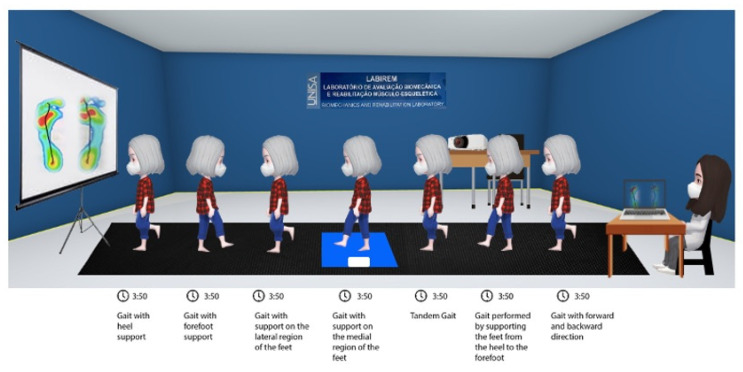
Gait Training Intervention with visual feedback in older women.

The complete description of the intervention protocol, including exercise parameters (2 sessions/week individually; beginner or advanced repetition schemes; progression based on sensory–motor level, task difficulty, fatigue, and pain) and the criteria for each intervention phase, is presented in Table 1, Table 2 and Table 3 (Figure 2 and Figure 3). At the end of the study, and in accordance with ethical guidelines, participants in the control group were offered the opportunity to complete an identical 8-week exercise program [38].

### 2.6. Outcome Measures

A physical therapist (researcher 4) who was blinded to group allocation conducted all assessments. The initial evaluation included the collection of personal information, anthropometric measurements, and all clinical, functional, and biomechanical outcomes. After the baseline assessment (T0), all participants were scheduled for post-intervention assessments (T8) [38].

### 2.7. Primary Outcomes

The International Society for Osteoarthritis Research states that the WOMAC questionnaire pain score should be chosen as the primary outcome in clinical trials. Another point established by the association is the functionality of these patients, also verified by the rigidity and function of activities of daily living in the WOMAC questionnaire, but also by the Lequesne Algofunctional questionnaire, both referenced in clinical trial studies, and with sensitivity to verify the changes and results of intervention programs. Thus, pain scores from the WOMAC questionnaire were used, as well as pain intensity verified by the Visual Analogue Scale (VAS), and functionality verified through both questionnaires: WOMAC and Lequesne [38,39].

### 2.8. Secondary Outcomes

As secondary outcomes, we assessed fall risk using the FRAQ-Brazil questionnaire, functional performance through the Six-Minute Walk Test and the Timed Up and Go Test, static balance, plantar load distribution during gait, and patient acceptability of the intervention protocol [38,39].

### 2.9. Clinical and Functional Assessment Protocol

This process was conducted at baseline (T0) and after two months of intervention (T8), following completion of the 16 exercise sessions. Clinical evaluation included radiographic examination to confirm osteoarthritic involvement according to the Kellgren and Lawrence criteria, followed by clinical confirmation of the knee osteoarthritis diagnosis by the attending physician. Pain intensity was assessed using the Visual Analogue Scale (VAS), where 0 indicates no pain, and 10 represents the worst imaginable pain, measured on a 10-cm line [38,41,42].

The functional assessment included the WOMAC (Western Ontario and McMaster Universities Osteoarthritis Index), the Lequesne Algofunctional Questionnaire—specific for knee OA—and the FRAQ-Brazil (Falls Risk Awareness Questionnaire), which assesses older adults’ perception of fall risk. In addition, the six-minute walk test and the Timed Up and Go test were administered to evaluate functional mobility and walking capacity. To evaluate the intervention, we administered the Acceptability, Appropriateness, and Feasibility Questionnaire. The WOMAC assesses three domains—pain, physical function, and joint stiffness—over the previous 72 h, through 24 items scored from 0 to 5, with higher scores indicating worse symptom severity [40]. The Lequesne Algofunctional Index consists of three sections (pain or discomfort, maximum walking distance, and activities of daily living), with scores ranging from 0 (no impairment) to 24 (extremely severe impairment). The translated and validated Brazilian version of the scale was used in the present study [43,44,45].

The FRAQ-Brazil questionnaire (Falls Risk Awareness Questionnaire) was used to assess fall-risk awareness in individuals aged 65 years and older. This instrument was originally developed at the University of Alberta, Canada, and later adapted for use in Brazil by Lopes and Trelha [46]. It comprises 25 multiple-choice questions, with total scores ranging from 0 (minimum) to 32 (maximum); higher scores indicate greater awareness of fall risk [46].

The six-minute walk test was used to assess the maximum distance (cm) that participants could walk within six minutes. This test evaluates functional exercise capacity by measuring the total distance covered during the allotted time. All participants were instructed to walk as fast and as far as possible throughout the six-minute period [47,48].

The Timed Up and Go Test (TUG) was applied to assess physical performance during walking and dynamic balance. The test measures the time required for the participant to stand up from a seated position, walk three meters to a marked point, turn around, return along the same path, and sit down again with their back against the chair. In terms of classification, times between 11 and 20 s are considered normal for frail older adults or individuals with disabilities. For values greater than or equal to 20 s, losses in physical performance and balance are considered, requiring appropriate interventions [38]. Furthermore, the intervention evaluation questionnaire was applied, divided into three domains: the first evaluates the measure of acceptability of the intervention (AIM), the second domain evaluates the measure of adequacy of the intervention (IAM), and the third and final domain evaluates the measure of intervention feasibility. Response options are given for each domain, and the score ranges from completely disagree to completely agree.

### 2.10. Biomechanical Assessment Protocol

For the biomechanical assessment of static balance, a pressure platform (Loran^®^, Sensor Medica Inc., Rome, Italy) was used. Participants stood in a bipedal stance with eyes open and arms relaxed alongside the body, maintaining a quiet upright posture for 60 s. Foot positioning was standardized at shoulder width (7 cm between the feet) and aligned. The variables analyzed included center of pressure (COP) displacement, anteroposterior (AP) oscillation, mediolateral (ML) oscillation, and COP velocity.

In the biomechanical assessment of plantar pressure distribution during gait, a pressure platform (Loran^®^, Sensor Medica Inc., Rome, Italy) was used, measuring 3240 mm in length, 620 mm in width, 20 mm in height, and weighing 29 kg. The equipment contains resistive pressure sensors homogeneously distributed at a density of 4 sensors/cm^2^. The platform was connected to a desktop computer for data acquisition, with recordings collected at a sampling frequency of 100 Hz. The force platform was embedded and fully aligned with the walkway, ensuring that all steps were performed on the same level. Cadence was monitored—but not controlled—using a metronome, ranging between 70 (baseline) and 100 steps per minute (post intervention). Additionally, participants underwent a brief habituation period before data collection to familiarize themselves with the environment and to reach their natural gait pattern. After a habituation period, three valid trials were collected. This procedure ensured that walking was continuous and uninterrupted when they stepped on the platform. The stance phase was evaluated bilaterally; however, only the step that fully contacted the platform during each timed trial was used for analysis to avoid contamination from partial or transitional foot contacts. Approximately 12 steps were acquired per participant, and the mean value was used for statistical analysis.

The older women were familiarized with the testing environment and equipment to minimize any reactive effects. After this adaptation period, participants walked on a flat synthetic rubber walkway over a distance of 20 m. The steps occurring within the intermediate 10-m segment were timed and considered valid for analysis, resulting in approximately 12 captured steps obtained over six walking trials, with foot contact on the pressure platform in each pass [38,39]. For data processing, a 4th-order Butterworth digital filter with a cutoff frequency of 6 Hz was applied to reduce noise while preserving the essential characteristics of the signal.

The plantar pressure variables analyzed and measured were: (1) Maximum Peak Pressure value per selected area: representing the maximum pressure value (expressed in kPa); (2) Maximum Mean Pressure: representing the average value of the maximum pressure (expressed in kPa), and (3) Contact area: representing the area in which the sensors were activated (pressed) in each step (expressed in cm^2^). All plantar pressure variables were analyzed in four plantar areas of the feet: medial and lateral rearfoot (30% of foot length), midfoot (30% of foot length), and forefoot and toes (40% of foot length) [38,39].

### 2.11. Statistical Analysis

The minimal clinically important difference (MCID) for the WOMAC pain subscale (1.5–2.0 points) was used to guide the original sample size estimation. To accommodate an anticipated dropout rate of approximately 15%, a total of 47 participants were enrolled. Of these, 40 participants (85%) completed all assessments and comprised the completer sample used for the primary and secondary efficacy analyses, thereby matching the effective sample size assumed in the power calculation. The missing data were imputed as appropriate, although no dropouts occurred. Per protocol, analysis included only patients who attended at least 90% of the sessions and completed the follow-up in the allocated intervention group.

Data normality and homogeneity of variance were assessed using the Shapiro–Wilk and Levene tests, respectively. After confirming normal distribution, parametric tests were applied to the outcome variables. A two-way mixed-model repeated-measures ANOVA was performed to assess differences between groups and intervention, followed by Tukey post hoc tests. This study involved two independent groups (EG and healthy controls) assessed at two different time points (pre- and post-intervention). This structure is best analyzed using a two-way mixed-design ANOVA, which allows simultaneous testing of: Main effect of group (differences between EG and CG, regardless of time); Main effect of time (changes over time, regardless of group), and Group × time interaction (whether the change over time differs between groups). Effect sizes and confidence intervals were reported using Cohen’s d, with values of 0.2, 0.4–0,7, and 0.8–1.0 being considered small, medium, and large effect sizes, respectively. In addition, the minimal clinically important difference (MCID) was considered, based on the calculation of the clinical effect size. A significance level of 5% was adopted for all analyses. Statistical procedures were conducted using SPSS version 17.0 (SPSS Inc., Chicago, IL, USA).

## 3. Results

Initially, 80 older women volunteered to participate in the study. Of these, 29 were excluded after one month of intervention due to a confirmed diagnosis of COVID-19, and 11 were excluded for other reasons (e.g., relocation, living too far from the intervention site, or schedule incompatibility). Among the participants who completed the intervention, adherence was high, with a mean attendance rate of 95% per session. The main reasons for missed sessions included scheduling conflicts and transportation difficulties. No adverse events associated with the intervention were reported. The proportion of missing data per outcome was 5%.

In total, 40 older women participated and completed the proposed intervention program, 20 with OA knee and 20 older controls (Figure 1). The intervention groups, OAG and CG, did not differ in relation to age, mass, height, BMI, and physical activity practice before and after two months of the intervention program, as seen in Table 4.

The older women in the intervention group with knee OA demonstrated reductions in knee and foot pain, as well as knee edema, along with improved knee functionality from pre- to post-intervention, as measured by the WOMAC and Lequesne questionnaires. Effect sizes ranged from moderate to high, demonstrating clinical significance that exceeded the established MCID values (Table 5).

**Figure 4:**
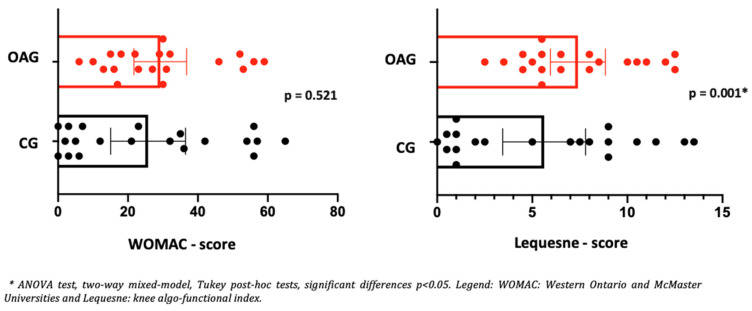
Means and *p*-values for comparison of the WOMAC and Lequesne total score between the groups of older women with OA knee (OAG) and older controls without knee osteoarthritis (CG) after two months.

In the control group of older women, no significant changes were observed in the clinical and functional aspects of the knee, with a low effect size for improving pain, edema, and knee functionality (Table 5). Regarding the functional aspects verified by WOMAC between the groups (OAG and CG), after two months of intervention, there was no statistical difference between the groups of older women. However, the OAG presented increased functionality (Lequesne questionnaire) when compared to CG (Figure 4). These findings tend to reveal the improvement of the intervention program in relation to control, showing it to be a pragmatic strategy to alleviate symptoms and improve functionality in OA knee (Figure 4).

**Figure 5:**
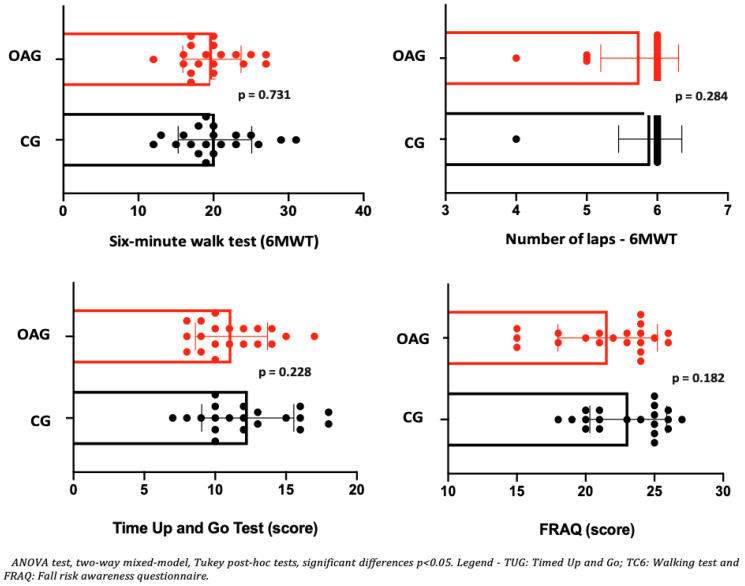
Means and *p*-values for comparison of the six-minute walk test (6MWT), balance (TUG), and perceived risk of falls (FRAQ) between groups of older women with OA knee (OAG) and older controls without knee osteoarthritis (CG) after two months.

Regarding aspects of dynamic balance (TUG), it can be observed that the intervention groups in older women with OA knee and control, when comparing pre and post-intervention, showed an increase in balance (TUG), with clinical significance by larger effect size for the OA knee group (d = 1.1) when compared to the control group (d = 0.68). In the walking test (6MWT), both groups (OA knee and control) showed an increase in walking distance and number of laps performed after the intervention period (Table 6). Regarding the perception of risk of falls (FRAQ), an increase was observed after the intervention in older women with OA knees and in control older women, with clinical significance (exceeding the MCID) indicated by a high effect size. In the comparison between the groups: OA and CG, after two months of intervention, no differences were observed for the six-minute walk test (6MWT), balance (TUG), and perception of risk of falls (FRAQ) in the OA when compared to the CG (Figure 5), showing the efficiency of the intervention protocol in older women with OA knee, since this group no longer showed differences in functional limitations from the control older women.

Regarding the parameters of plantar pressure distribution, it can be observed that before and after the intervention program, the OA knee showed a reduction in the contact area in the medial and lateral rearfoot region. In relation to peak pressure and maximum force in the midfoot and the medial and lateral rearfoot regions, a reduction in plantar overload was observed, indicating a more efficient motor strategy for controlling the center of gravity during the heel-strike and mid-stance phases. This adaptation contributes to a more balanced distribution of forces and facilitates the propulsion phase of gait. In the control group, no improvement in contact area was detected before or after the intervention; however, a reduction in peak pressure and maximum force was observed in the rearfoot (Table 7). Effect sizes ranged from small to moderate, and the magnitude of change exceeded the established MCID values, supporting the clinical relevance of the findings.

**Figure 6:**
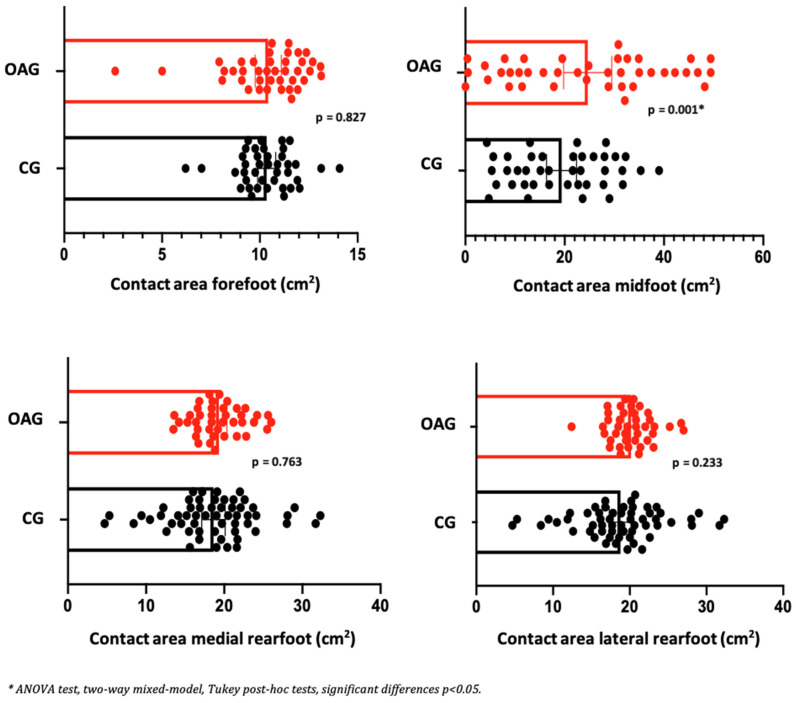
Means and *p*-values for comparison of the contact area in the foot region (forefoot, midfoot, and medial and lateral hindfoot) between the groups of older women with OA knee (OAG) and older controls without knee osteoarthritis (CG) after two months of the intervention protocol.

**Figure 7:**
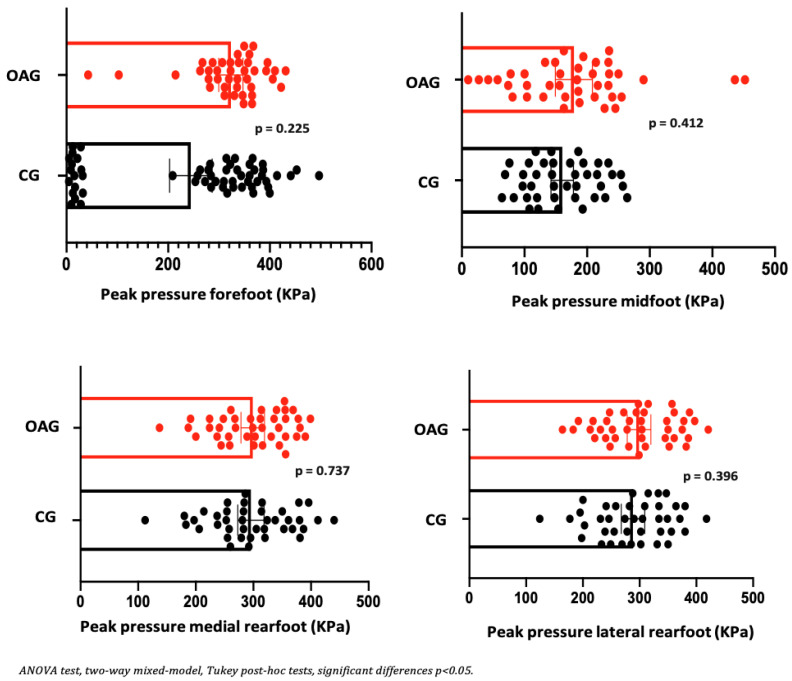
Means and *p*-values for comparison of the peak pressure in the foot region (forefoot, midfoot, and medial and lateral hindfoot) between the groups of older women with OA knee (OAG) and older controls without knee osteoarthritis (CG) after two months of the intervention protocol.

**Figure 8:**
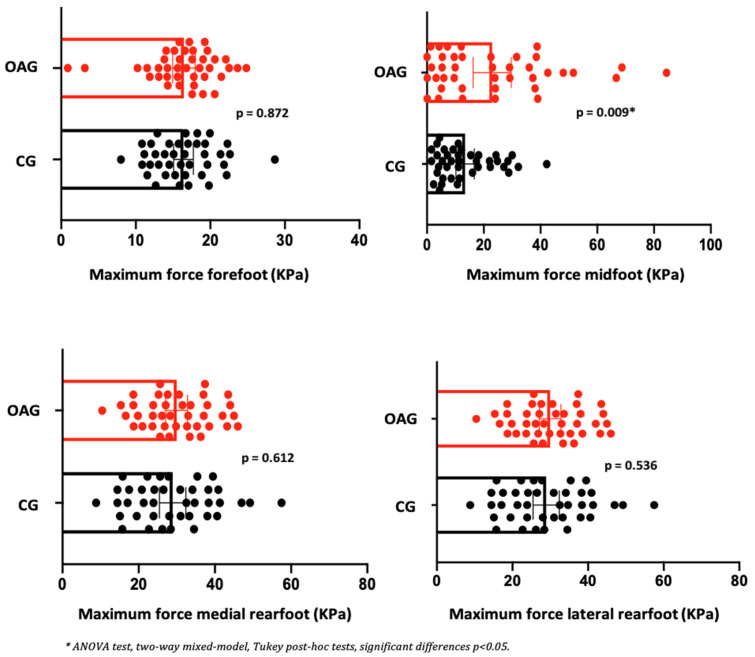
Means and *p*-values for comparison of the maximum force in the foot region (forefoot, midfoot, and medial and lateral hindfoot) between the groups of older women with OA knee (OAG) and older controls without knee osteoarthritis (CG) after two months of the intervention protocol.

In the comparison between groups (OAG and CG), after the intervention period, the older women with knee osteoarthritis demonstrated greater contact area and maximum force in the midfoot region compared with the control group, showing a better load-bearing strategy in this region, favoring the mid-stance phase of gait. The peak pressure, in all regions of the feet, did not present significant differences, showing the efficiency of the intervention program for better dissipation of the plantar load when compared to the older controls (Figure 6, Figure 7 and Figure 8).

In Table 8, it can be seen that older women with and without OA knee showed excellent acceptability, as well as appropriateness, and feasibility for both intervention groups: OAG and CG, demonstrating the intervention to be a pragmatic therapeutic strategy for applicability in public healthcare sectors.

## 4. Discussion

The purpose of this study was to evaluate the therapeutic effects of a lower-limb resistance training program combined with balance exercises and gait training with visual feedback on the clinical, functional, and biomechanical outcomes of older women with and without knee osteoarthritis. The main findings indicate that, after two months of intervention, older women with knee OA showed reductions in pain and knee edema, improvements in functional performance (WOMAC and Lequesne), enhanced balance (TUG), and better awareness of fall risk. Additionally, during gait with visual feedback, these participants demonstrated reduced overload in the midfoot and medial and lateral rearfoot regions, whereas the control group showed reductions only in rearfoot plantar overload (medial and lateral) after the intervention.

Kasicki et al. [49], in a recent systematic review, reported that multicomponent training programs—integrating strength, balance, and aerobic exercises—are superior to single-component interventions in preventing falls and enhancing functional outcomes in older adults. Our findings align with this evidence and further extend it. Specifically, beyond employing a multicomponent exercise framework, our study incorporated gait training with visual feedback, targeting the lower-limb strength demands that often constrain gait performance in this population. This additional component appears to have contributed to the observed improvements across clinical, functional, and gait parameters, suggesting that visual-feedback-based gait training may represent a valuable complement to traditional multicomponent exercise protocols.

According to Wan et al. [50], a comprehensive and effective gait retraining program can represent a practical and self-administered approach to slow the progression of knee osteoarthritis and improve the quality of life in patients with this condition. Studies focused on sports, especially running, have shown that gait retraining with visual feedback can reduce plantar overload by increasing the activation of proprioceptive sensors, improving the sensorimotor response to mimic the impact forces received [51].

Another systematic review studies reveal moderate to high evidence on general or combined therapeutic exercises to reduce pain and increase knee functionality, as well as motor agility training for functional improvement [52,53]. In the present study, the magnitude of improvement reached clinically meaningful thresholds—for example, WOMAC pain improvements aligned with established MCID ranges (1.5–2.0 points), as well as the improvement in the WOMAC total score and the TUG and 6MWT tests were compatible with meaningful functional gains described in older adults, showed a large effect size for reducing pain and edema and increasing knee functionality in older women with OA.

Considering muscular resistance training, functional performance associated with joint mobility, several studies have shown the efficiency of these rehabilitation programs for the conservative treatment of older women with OA knee lasting 8 to 12 weeks [23,52,53,54,55]. The distinguishing feature of the present study was demonstrating that, within a relatively short intervention period of two consecutive months (8 weeks; 16 sessions), a program combining muscular resistance exercises for the quadriceps, triceps surae, and intrinsic foot muscles with balance and gait training incorporating visual feedback was effective in improving clinical and functional outcomes in older women with and without knee osteoarthritis. These findings highlight the protocol as a pragmatic therapeutic strategy capable of reducing symptoms and functional limitations in this population, further supported by participants’ reports of high acceptability and feasibility. Strength training should be considered to increase muscle function and dynamic balance in older women, whereas multicomponent training should be considered to increase functional capacity and gait ability in this population [56]. This rationale may explain the benefits of combining gait retraining with visual feedback with a strength and balance exercise program to improve plantar load distribution, increase functionality, and reduce the risk of falls.

In this partial line of reasoning, other studies carried out by Raposo, Ramos and Cruz [33] and Vincent and Vincent [25], showed that lower limb muscle strengthening exercise programs (concentric and eccentric) associated with aerobic exercises, between 8 and 16 weeks (2–3 weekly sessions lasting 45 min each session) are effective in improving pain and functionality of individuals with OA knee. In addition to these benefits, the protocol also enhanced balance and contributed to better fall prevention in older adults [57]. In the current study, the intervention protocol used eccentric muscular strength training combined with proactive and reactive balance training exercises and gait training with visual feedback, and the results showed significant improvements in the reduction of pain and edema, and an increase in the physical function of older women with OA of the knee, even after a short period of time (2 consecutive months). These findings were corroborated in a recent systematic review by Turne et al. [58], who observed that muscular resistance training, when combined with physical exercise, can improve pain and/or physical function in individuals with OA of the knee.

Another study, carried out by Chen et al. [59], investigated home-based training over 12 weeks for older people with OA of the knee. The training program included lower-limb resistance exercises and balance training with changing directions. The authors concluded that the intervention program was effective in reducing pain and joint stiffness and in improving knee functionality in patients with OA. A key contribution of the present study was the integration of gait training with visual feedback into a program of muscle strengthening and balance exercises, delivered individually in a clinical laboratory setting and in full compliance with COVID-19 safety protocols. This combined approach resulted in meaningful improvements in the motor and functional strategies of older women with knee osteoarthritis. The visual stimulus used during gait training may have strengthened the integration of visual, sensorimotor, and proprioceptive cues, supporting the adoption of more adaptive cognitive–motor strategies. By augmenting the quality and quantity of sensory feedback available during task performance, this approach likely amplified neuromotor afferent signaling, contributing to more accurate motor control and more efficient gait execution in the older women’s strategy to increase neuromotor afferents in response to the exercises.

Some studies have shown the efficiency of motor strategies with therapeutic muscular strength exercises of the trunk and lower limbs (hips and knees) associated with balance training to reduce pain and improve function in patients with OA knee [25,34], while a quadriceps and hamstring muscle strengthening program, together with proprioception and balance training, obtained the same benefits in older women with OA knee [34,60]. An important finding of this study was to verify that a muscular resistance protocol for quadriceps and hamstrings with reactive and proactive balance training associated with gait training with visual feedback, in addition to improving clinical and functional aspects, increased balance and perception of the risk of falls, creating better motor strategies to alert to the risk of falling. This finding can help reduce falls and improve knee function during daily activities, such as walking, in older women with OA of the knee. A central mechanism in our study relates to the use of real-time visual feedback during gait: participants viewed screen-projected images of foot rolling and peak plantar pressure, allowing immediate self-correction. This aligns with current evidence showing that real-time visual biofeedback enhances sensorimotor integration, improves foot-placement accuracy, improves control of the center of mass, and redistributes plantar loads in older adults [49,51].

Another important finding obtained was the redistribution of pressure plantar over the forefoot, midfoot, and rearfoot (lateral and medial) in older women with OA knee, proving to be a therapeutic strategy for adjusting and controlling the center of gravity over the support of the feet, dissipating the vectors of impact forces received during walking, and consequently, on the knee, favoring better body balance. Zhang et al. [61] compared the plantar loads of older women with OA and control (mild to moderate degree) during walking, and observed an increase in peak pressure and maximum force in the midfoot and forefoot regions when compared to control older women. According to Lidtke et al. [62], increased lateral plantar load on the feet and worsened balance control can increase impact forces on knees with OA during walking, and progressively worsen pain and functional limitations. The difference in this study was to apply an intervention protocol and verify a reduction in peak pressure over all areas of the feet after the intervention protocol. It is possible that the fact that the older women performed a targeted gait training protocol, individually and with stimulation from visual feedback, is a better-suited strategy for dissipating the plantar loads received by the feet in contact with the ground, mitigating the force vectors imposed on the knee with OA.

The explanation for this rationale comes from specific studies with visual biofeedback training (mirror or video) associated with the exercise and balance training program for older women with OA knee, in which the authors highlight the efficiency of these protocols in improving balance and functional knee mobility during walking [26,27], as well as training of gait phases associated with visual feedback (mirror and video) to increase walking activity and minimize mechanical loads on the knee [63]. The difference in this study was to associate visual feedback through the mirroring of the computer screen, with images of the lower limbs during walking, on the wall in front of the patient, facilitating corrections of foot support during gait training.

Another study by Zukowski et al. [64] on the effects of treadmill training with virtual reality (semi-immersive) on aspects of gait and cognition in older people in relation to conventional treadmill training, in a single session, showed no differences in relation to gait and cognition between the virtual reality and conventional training groups, however in terms of speed there was improvement in both groups in single and dual-task walking, which is associated with a reduced risk of falls. In the present study, an intervention protocol that combined ground gait training with visual feedback for a period of more than 2 months proved to be beneficial in patients with OA knee, reducing the effects of the clinical framework and disease progression, due to the better redistribution of plantar load achieved by foot rolling training with visual feedback stimulation, which enabled the older women to correct their plantar support. Considering clinical implementation, these findings support the feasibility of visual-feedback–guided gait and balance training in supervised, community, or tele-rehabilitation environments. Post-pandemic studies have shown that visual-feedback systems—ranging from screen-based projection to smartphone or telehealth platforms—are viable for remote or hybrid rehabilitation while maintaining clinical effectiveness [64].

The main limitation of this study was the difficulty in recruiting participants during the COVID-19 pandemic, due to consecutive periods of social isolation that restricted the participation of a larger number of older women with and without OA knee in the intervention protocol. Additionally, the study did not include a long-term follow-up period, which limits the ability to determine whether the clinical, functional, and biomechanical improvements observed were sustained over time. The volunteer-based recruitment strategy may also have introduced self-selection bias, as individuals who agree to participate in structured exercise programs tend to be more motivated and potentially more responsive to rehabilitation interventions. However, it is worth highlighting that even though the COVID-19 pandemic may have influenced motivation, participation, and psychological well-being among the older women, the intervention was rated highly in terms of acceptability, appropriateness, and feasibility, which likely contributed to the strong adherence observed throughout the treatment. Furthermore, the sample consisted exclusively of older women, which restricts the generalizability of the findings to men or mixed-gender populations. Future studies should incorporate longer follow-up periods, broader recruitment methods, and more diverse samples to enhance the external validity of the results.

## 5. Conclusions

The intervention protocol—which included muscle resistance training, reactive and proactive balance exercises, and gait training with visual feedback—proved effective over a period of two consecutive months in reducing pain and swelling and improving knee function, balance, walking distance, and perceived fall risk in older women with knee osteoarthritis. Improvements in functional outcomes were also observed in the age-matched comparison group of women without knee osteoarthritis, indicating that the protocol benefits older adults regardless of OA status.

During gait with visual feedback, the protocol contributed to minimizing plantar load on midfoot and rearfoot (medial and lateral) in women with knee osteoarthritis. High acceptability and feasibility were reported in both groups, supporting the practicality of implementing this protocol in clinical or community-based rehabilitation settings. Given its structured design, low cost, and adaptability, the protocol can also be translated to tele-rehabilitation formats, particularly in post-pandemic contexts where remote monitoring and home-based guidance remain increasingly relevant.

## Figures and Tables

**Table 1:** Intervention Protocol with Muscular Resistance Training and Static Balance: description, execution, and parameters of the exercises.

Exercises	Exercise Variables	Recommendations
Muscle resistance and static balance training	Protection equipment	Disposable masks, Face shield, Disposable gloves, and Alcohol gel
	Support base	Stable and Unstable: Bipedal—Unipodal—Semi-tandem—Tandem (Figure 1)
	Surface	Stable—Mat
	Sensory	Eyes open; Eyes closed
	Muscle groups	Knee: quadriceps, hamstrings, tibialis, fibular, and triceps surae; Foot: flexors, extensors, and intrinsic musculature
	Intensity	Defined by difficulty level, fatigue, and number of repetitions
	Movement speed	Slow Speed (concentric phase 2 s and eccentric phase 4 s);
	Contraction speed	Moderate Speed (concentric phase 1 s and eccentric phase 2 s)
Intensity Parameters	Frequency	2 sessions/week, individually
	Repetitions	Beginner: 10–15 (moderate stamina) Advanced: 8–12 (high stamina)
	Rest interval	2 min every five repetitions
Progression Parameters	Progression parameters	No Pain or Muscle Fatigue
	Duration	15 min
	Foot condition	No pain sensation

**Table 2:** Intervention Protocol with Dynamic Balance Training (sensory and motor), Reactive and Proactive: description, execution, and parameters of the exercises.

Exercises	Exercise Variables	Recommendations
Dynamic Balance Training	Protection equipment	Disposable masks, Face shield, Disposable gloves, and Alcohol gel
	Support base	Stable and Unstable: Bipedal—Unipedal (Figure 2)
	Surface	Stable—Mat; Unstable—Mattress
	Foot position	Shifting weight on the toes and heel
	Intensity	Defined by difficulty level, fatigue, and number of repetitions
	Frequency	2 sessions/week, individually
Intensity Parameters	Repetitions	Beginner: five times with 30 s on each side;
		Advanced: ten times with 30 s on each side.
	Rest interval	2 min every five repetitions
Progression Parameters	Progression parameters	Acquire the skill of base support, sensory, and motor exercises to evolve to reactive and proactive exercises
	Duration	10 min
Balance training with sensory exercise	Support base	Balance disc
	Foot position	Bipedal
	Surface	Flat mat made of flexible rubber fabric
Balance training with motor exercise	Walk with obstacles	Normal; Tandem; Lateral
	Movement speed	Slow; Fast
	Sensory	Eyes open; Eyes closed
Reactive Exercise	Disturbances monitored by the physical therapist	At the level of the shoulder, trunk, hip, and ankle joint segments
Proactive Exercise	Activities of Daily Living (ADLs)	Sit and get up from a chair with bipedal support
	Foot condition	Oscillatory support of the plantar base
	Protection equipment	Disposable masks, Face shield, and Disposable gloves

**Table 3:** Intervention Protocol with Gait Training with Visual Feedback: description, execution, and parameters of the exercises.

Exercise	Exercise Variables	Recommendations
Gait training with visual feedback	Protection equipment	Disposable masks, Face shield, Disposable gloves, and Alcohol gel
	Gait with support and displacement	Heel; forefoot; side edge; Medial Edge; Tandem; Displacement from heel to forefoot; Forward and backward direction (Figure 3)
	Surface	Stable—Mat (made of flexible rubber fabric)
	Rolling of the feet in the phases of the gait	Load bearing in the initial phases (heel support), intermediate phase (lateral midfoot support), and propulsion phase (lateromedial forefoot support)
Intensity Parameters	Intensity	Walk 112 m (round trip from exercises)
	Frequency	2 sessions/week individually
	Repetitions	Beginner: two times in each gait training
Progression Parameters	Progression parameters	Support of the feet in the different phases of gait (initial, intermediate, and propulsion contact) with balance disturbance
	Duration	15 min
Gait training with speed	Movement speed	Slow; Fast
	Foot condition	Distribution of plantar load on the different regions of the feet

**Table 4:** Mean, standard deviation, percentage, and comparison of anthropometric aspects and percentage of physical activity practice before (pre) and after (post) the therapeutic intervention protocol of older women with knee osteoarthritis (OAG) and older controls without knee osteoarthritis (CG).

Variables	OAG (n = 20)			CG (n = 20)		
	Pre	Post	*p* *	Pre	Post	*p* *
Age (years)	67.4 ± 4.9	67.5 ± 5.2	0.540	68.1 ± 6.4	68.4 ± 6.2	0.210
Weight (Kg/cm^2^)	75.4 ± 13.3	75.6 ± 12.8	0.586	66.6 ± 12.8	68.0 ± 12.9	0.206
Height (cm)	1.59 ± 0.7	1.60 ± 0.7	0.989	1.56 ± 0.8	1.55 ± 0.8	0.803
BMI (Kg/cm^2^)	29.9 ± 5.2	29.5 ± 4.0	0.578	27.4 ± 4.5	28.1 ± 4.8	0.124
Practice of physical activity—walking (min/week)	10% (S)	30% (S)	-	60% (S)	40% (S)	-
	90% (N)	70% (N)		40% (N)	60% (N)	

* ANOVA test, two-way mixed-model repeated-measures, Tukey post hoc tests, significant differences *p* < 0.05. Significant differences between moments: pre- and post-intervention. Legend: (S): yes; (N): no.

**Table 5:** Mean, standard deviation, and comparison of clinical aspects before (pre) and after (post) the therapeutic intervention protocol among older women with knee osteoarthritis (OAG) and older controls without knee osteoarthritis (CG).

Clinical Variables	OAG Knee (n = 20)				CG (n = 20)			
	Pre	Post	d **	*p* *	Pre	Post	d **	*p* *
Knee pain (with)	8.5 ± 1.0	5.6 ± 2.8	0.88	0.002 *	3.1 ± 1.4	2.5 ± 1.6	0.39	0.374
Feet pain (with)	6.4 ± 3.0	4.5 ± 1.9	0.75	0.001 *	6.0 ± 3.4	5.0 ± 3.0	0.31	0.120
Knee swelling R (with)	37.8 ± 4.5	36.7 ± 4.0	0.25	0.020 *	37.1 ± 3.2	37.2 ± 2.8	0.03	0.856
Knee swelling L (with)	38.0 ± 4.6	36.9 ± 4.0	0.26	0.014 *	37.5 ± 3.4	36.9 ± 3.1	0.18	0.387
WOMAC (score)	48.7 ± 22.7	29.9 ± 17.4	0.92	0.001 *	23.4 ± 16.5	25.7 ± 17.9	0.13	0.621
Lequesne (score)	11.0 ± 3.5	7.4 ± 3.1	1.0	0.001 *	6.1 ± 4.9	5.6 ± 4.5	0.10	0.556

* ANOVA test, two-way mixed-model repeated-measures, Tukey post hoc tests, significant differences *p* < 0.05. Significant differences between moments: pre- and post-intervention. ** Cohen’s d test to verify the effect of the intervention. Legend: WOMAC: Western Ontario and McMaster Universities and Lequesne: knee algofunctional index.

**Table 6:** Mean, standard deviation, and comparison of functional aspects before (pre) and after (post) the therapeutic intervention protocol of older women with knee osteoarthritis (OAG) and older controls without knee osteoarthritis (CG).

Functional Variables	OAG Knee (n = 20)				CG (n = 20)			
	Pre	Post	d **	*p* *	Pre	Post	d **	*p* *
Time Up and Go –TUG (sec.)	14.0 ± 2.8	11.1 ± 2.5	1.3	0.001 *	13.1 ± 3.5	11.0 ± 3.2	0.62	0.002 *
Walking test-6MWT (minutes)	17.2 ± 6.2	19.8 ± 3.8	0.50	0.011 *	17.6 ± 4.8	20.2 ± 4.8	0.54	0.025 *
Walking test-6MWT (laps)	5.5 ± 1.2	6.0 ± 0.5	0.55	0.018 *	5.7 ± 1.0	6.0 ± 0.4	0.39	0.012 *
Fall risk awareness questionnaire—FRAQ (sec.)	20.1 ± 4.1	21.6 ± 3.6	0.38	0.012 *	20.7 ± 2.5	23.1 ± 2.8	0.90	0.001 *

* ANOVA test, two-way mixed-model repeated-measures, Tukey post hoc tests, significant differences *p* < 0.05. Significant differences between moments: pre- and post-intervention. ** Cohen’s d test to verify the effect of the intervention. Legend—TUG: Timed Up and Go; TC6: Walking test; and FRAQ: Fall risk awareness questionnaire.

**Table 7:** Mean, standard deviation, and comparison of the biomechanical aspects of the distribution of foot plantar pressure during walking before (pre) and after (post) the therapeutic intervention protocol of older controls without knee osteoarthritis (CG).

Biomechanics Variables	Foot Regions	OAG Knee (n = 20)				CG (n = 20)			
		Pre	Post	d **	*p* *	Pre	Post	d **	*p* *
Contact Area (cm^2^)	Forefoot	10.8 ± 1.8	10.4 ± 2.0	0.21	0.275	10.4 ± 1.2	10.3 ± 1.4	0.07	0.755
	Midfoot	26.9 ± 11.8	24.6 ± 15.1	0.17	0.110	19.5 ± 10.5	19.3 ± 9.4	0.02	0.803
	Medial hindfoot	20.4 ± 2.9	19.3 ± 3.2	0.36	0.032 *	19.8 ± 2.9	19.1 ± 2.5	0.25	0.084
	Lateral hindfoot	21.0 ± 2.6	20.1 ± 2.8	0.33	0.012 *	20.4 ± 2.7	19.4 ± 2.7	0.37	0.056
Peak Pressure (KPa)	Forefoot	313.5 ± 65.7	323.5 ± 74.5	0.14	0.329	321.0 ± 63.7	341.2 ± 59.1	0.32	0.090
	Midfoot	193.0 ± 74.9	178.7 ± 82.5	0.18	0.029 *	162.8 ± 59.2	160.4 ± 56.0	0.04	0.833
	Medial hindfoot	310.7 ± 75.3	298.9 ± 63.7	0.17	0.004 *	300.8 ± 71.7	295.7 ± 70.5	0.17	0.002 *
	Lateral hindfoot	310.3 ± 66.1	298.8 ± 76.1	0.16	0.001 *	290.1 ± 68.6	282.3 ± 64.5	0.11	0.001 *
Maximum Force (N/BW)	Forefoot	16.3 ± 4.5	16.4 ± 4.7	0.02	0.876	15.8 ± 3.4	16.5 ± 4.1	0.18	0.465
	Midfoot	24.8 ± 17.5	22.9 ± 10.9	0.13	0.040 *	14.0 ± 5.6	13.5 ± 5.7	0.08	0.796
	Medial hindfoot	33.5 ± 10.6	29.1 ± 7.8	0.47	0.033 *	32.1 ± 10.3	29.2 ± 9.5	0.30	0.001 *
	Lateral hindfoot	33.0 ± 9.7	29.9 ± 9.0	0.33	0.005 *	31.5 ± 9.7	27.4 ± 8.7	0.44	0.025 *

* ANOVA test, two-way mixed-model repeated-measures, Tukey post hoc tests, significant differences *p* < 0.05. Significant differences between moments: pre- and post-intervention. ** Cohen’s d test to verify the effect of the intervention.

**Table 8:** Description of the results of the acceptability, appropriateness, and feasibility of the therapeutic intervention protocol for older women with knee osteoarthritis (OAG) and older controls (CG).

	CG (n = 20)	OAG (n = 20)
**Measure of Intervention Acceptability (AMI), Med. (SD)**		
The intervention meets with my approval	4.89 (0.32)	4.74 (0.45)
The intervention is appealing to me	4.79 (0.71)	4.68 (0.48)
I like the intervention	4.89 (0.32)	4.79 (0.42)
I accept the intervention	4.95 (0.23)	4.84 (0.50)
**Total Score, Med. (SD)**	**4.88 (0.23)**	**4.76 (0.43)**
**Measure of Intervention Appropriateness (MAI), Med. (SD)**		
The intervention seems appropriate	4.84 (0.37)	4.74 (0.45)
The intervention seems adequate	4.89 (0.32)	4.68 (0.48)
The intervention seems applicable	4.79 (0.42)	4.37 (0.90)
The intervention seems to be a good option	4.89 (0.32)	4.68 (0.48)
**Total Score, Med. (SD)**	**4.86 (0.27)**	**4.62 (0.49)**
**Measure of Intervention Feasibility (VMI), Med. (SD)**		
The intervention seems implementable	4.74 (0.73)	4.58 (0.51)
The intervention seems possible	4.74 (0.74)	4.47 (0.77)
The intervention seems viable	4.84 (0.37)	4.68 (0.48)
The intervention seems easy to use	4.73 (0.45)	4.42 (0.69)
**Score Total, Med. (DP)**	**4.76 (0.51)**	**4.54 (0.49)**

## Data Availability

The original contributions presented in this study are included in the article. Further inquiries can be directed to the corresponding author.
